# A Multipathway Phosphopeptide Standard for Rapid Phosphoproteomics Assay Development

**DOI:** 10.1016/j.mcpro.2023.100639

**Published:** 2023-08-30

**Authors:** Brian C. Searle, Allis Chien, Antonius Koller, David Hawke, Anthony W. Herren, Jenny Kim Kim, Kimberly A. Lee, Ryan D. Leib, Alissa J. Nelson, Purvi Patel, Jian Min Ren, Paul M. Stemmer, Yiying Zhu, Benjamin A. Neely, Bhavin Patel

**Affiliations:** 1 Department of Biomedical Informatics, The Ohio State University, Columbus, Ohio, USA; 2 Pelotonia Institute for Immuno-Oncology, The Ohio State University Comprehensive Cancer Center, Columbus, Ohio, USA; 3 Mass Spectrometry Center, Stanford University, Stanford, California, USA; 4 YatiriBio, San Diego, California, USA; 5 BreakBio Corp, New York, New York, USA; 6 UC Davis Genome Center, Proteomics Core, University of California Davis, Davis California, USA; 7 Herbert Irving Comprehensive Cancer Center, Columbia University Medical Center, New York, New York, USA; 8 Cell Signaling Technology, Inc, Danvers, Massachusetts, USA; 9 Department of Pharmaceutical Sciences, Wayne State University, Detroit, Michigan, USA; 10 National Institute of Standards and Technology, Charleston, South Carolina, USA; 11 Thermo Fisher Scientific, Rockford, Illinois, USA

**Keywords:** proteomics, phosphorylation, phosphopeptide, mass spectrometry, targeted, data-independent acquisition, stable isotope label

## Abstract

Recent advances in methodology have made phosphopeptide analysis a tractable problem for many proteomics researchers. There are now a wide variety of robust and accessible enrichment strategies to generate phosphoproteomes while free or inexpensive software tools for quantitation and site localization have simplified phosphoproteome analysis workflow tremendously. As a research group under the Association for Biomolecular Resource Facilities umbrella, the Proteomics Standards Research Group has worked to develop a multipathway phosphopeptide standard based on a mixture of heavy-labeled phosphopeptides designed to enable researchers to rapidly develop assays. This mixture contains 131 mass spectrometry vetted phosphopeptides specifically chosen to cover as many known biologically interesting phosphosites as possible from seven different signaling networks: AMPK signaling, death and apoptosis signaling, ErbB signaling, insulin/insulin-like growth factor-1 signaling, mTOR signaling, PI3K/AKT signaling, and stress (p38/SAPK/JNK) signaling. Here, we describe a characterization of this mixture spiked into a HeLa tryptic digest stimulated with both epidermal growth factor and insulin-like growth factor-1 to activate the MAPK and PI3K/AKT/mTOR pathways. We further demonstrate a comparison of phosphoproteomic profiling of HeLa performed independently in five labs using this phosphopeptide mixture with data-independent acquisition. Despite different experimental and instrumentation processes, we found that labs could produce reproducible, harmonized datasets by reporting measurements as ratios to the standard, while intensity measurements showed lower consistency between labs even after normalization. Our results suggest that widely available, biologically relevant phosphopeptide standards can act as a quantitative “yardstick” across laboratories and sample preparations enabling experimental designs larger than a single laboratory can perform. Raw data files are publicly available in the MassIVE dataset MSV000090564.

## Highlights

• A multipathway phosphopeptide standard of 131 heavy-labeled phosphopeptides.
• Five labs produced harmonized results by reporting data as ratios to the standard.
• Harmonized ratios were significantly more consistent than intensity measurements.
• This standard acts as a quantitative “yardstick” across laboratories.
• The material is publicly available as a high-purity commercial standard.

## Graphical Abstract

**Figure:**
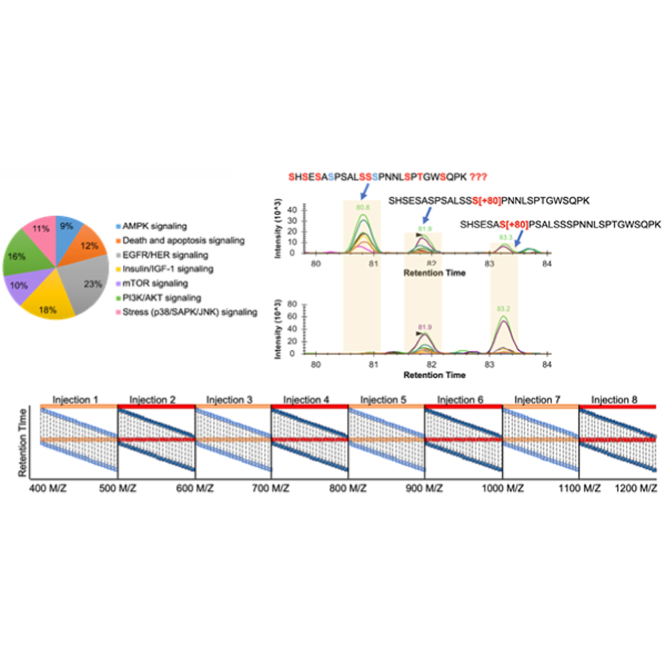

## In Brief

The Proteomics Standards Research Group developed a multipathway standard mixture of heavy-labeled phosphopeptides. This mixture contains 131 vetted phosphopeptides that cover biologically interesting phosphosites from seven different signaling networks. We characterized this mixture in five labs using data-independent acquisition. Despite different experimental processes, labs produced reproducible, harmonized datasets by reporting measurements as ratios to the standard. Our results suggest that widely available, biologically-relevant standards act as quantitative “yardsticks” across laboratories, enabling experimental designs larger than a single laboratory can perform.

Signaling through protein phosphorylation is akin to a molecular switch that regulates a wide variety of cellular activity, including cell metabolism, differentiation, and proliferation. Manipulating phosphorylation is the key to unlocking therapies for a wide variety of diseases (1) and several protein kinases are tractable drug targets (2). While a wide variety of biochemical tools are available for studying protein phosphorylation, including radioactive phosphorus and site-specific antibodies, mass spectrometry remains a powerful technique for tracking phosphorylation. When coupled with immobilized metal ion affinity chromatography (IMAC) (3, 4), metal oxide enrichment (*e.g.*, TiO2) (5), strong cation exchange (6), or antibody enrichment (7, 8, 9, 10, 11), tandem mass spectrometry (MS/MS) for phosphopeptides can globally quantify hundreds or thousands of phosphosites in a single injection. To that end, large-scale mass spectrometric analyses of the human phosphoproteome (12, 13, 14) have revealed hundreds of thousands of phosphorylated residues distributed across over half of the human proteome.

Most phosphoproteomics workflows using mass spectrometry make use of data-dependent acquisition (DDA) (15), where the most intense peptide precursors are selected for MS/MS fragmentation using dynamic exclusion to avoid measuring the same peptide multiple times. The reliance on making measurements based on different signal intensities in each sample results in stochastic sampling producing sparse datasets with many missing quantitative values (16). Systematic techniques, such as parallel reaction monitoring (PRM) (17) and data-independent acquisition (DIA) (18, 19) use preprogrammed MS/MS windows to monitor phosphopeptides. With PRM, researchers select specific peptide precursors and retention time windows to sample a limited number of targeted peptides with high fidelity. Conversely, DIA actively multiplexes peptide measurements by cofragmenting peptides within a wide precursor isolation window, resulting in proteome-wide quantification at the cost of signal interference. Due to systematic scanning, DIA has a demonstrated track record of producing quantitative measurements that are highly reproducible in multi-instrument (20) and multisite experiments (21). New methods using gas-phase fractionation (GPF) coupled with DIA (22, 23) seek to collect PRM quality data across the entire proteome using multiple injections of the same sample measuring different *m/z* ranges with small (approximately 2 *m/z*-wide) windows. Additionally, GPF-DIA can be used on sample pools to generate DIA-specific chromatogram libraries for improving the analysis of other standard DIA injections (24, 25) and this approach has been successfully applied to large-scale phosphoproteomics experiments (26).

Interpretation of phosphopeptides with PRM and DIA methods remains difficult, in part due to isobaric phosphosites where the same peptide can be phosphorylated at multiple residues (27, 28, 29). Combinatorial phosphopeptide libraries (30) or proteome-scale synthetic peptides (31, 32) can help improve data analysis or empirically identify interacting binding partners but are difficult to use as internal standards for quantifying endogenous peptides in unknown samples. Here, synthesized stable isotope labeled (SIL) phosphopeptide standards can be used to indicate endogenous signals specific to each site (33, 34, 35, 36, 37) because they share the same chemical properties (and thus the same retention time and fragmentation patterns) but with different ion masses. Additionally, spiked SIL phosphopeptides facilitate interlaboratory comparisons (38) and improve quantification across large-scale experiments (39, 40). Finally, SIL peptides can act as retention time standards to align spectrum libraries to a dataset (41) and aid in interpreting global phosphopeptide experiments.

Purified SIL phosphopeptides remain both expensive and challenging to produce at scale, rendering full proteome coverage with SIL standards impractical. Some publicly available, large-scale SIL phosphoproteomics standards have attempted to produce a reduced representation of the general phosphorylation state by focusing on monitoring selected highly observed peptides that produce easily measured signals (35, 38). In this study, we have developed a standard to monitor kinase signaling state by measuring common “hub and spoke” proteins where activation of these hubs can lead to massive changes in downstream signaling (11, 42). For example, AKT has as many as 150 substrates (43, 44), yet signal transduction is largely controlled by the phosphorylation at two sites: T308 and S473 (45). Monitoring “sentinel” phosphosites like these, which are closer to a wide variety of biological activity and functions, may produce a more refined interpretation of active signaling pathways than phosphosites in reduced-representation assays that are selected strictly because they are easy to observe (33). In this work, we present this multipathway phosphoproteomics SIL standard to measure key biologically relevant phosphosites in human proteins indicating specific kinase or pathway activity and demonstrate its utility in a multilab comparative analysis of a human-derived sample.

## Experimental Procedures

### Peptide Synthesis

All phosphopeptides were generated using PEPotec SRM Custom Peptide Libraries Synthesis Service (Thermo Fisher Scientific). Briefly, all peptides were synthesized in a crude format using the latest Fmoc solid-phase technology. Synthesized peptides were cleaved using a standard cleavage cocktail and suspended in 0.1% (volume fraction) TFA in 50% (volume fraction) acetonitrile in water. The molecular weight for each peptide was confirmed using a mass spectrometer equipped with a nanospray source. Heavy isotope-labeled amino acids used for synthesis are R (+10.008 Da), K (+8.014 Da), V (+6.014 Da), and A (+4.007 Da).

### HeLa Cell Culture

HeLa cells (American Type Culture Collection, CCL-2) were maintained as a monolayer (100% relative humidity, 95% air, 5% CO_2_ by volume) in Eagle's minimum essential medium (Corning 10–010) supplemented with, MEM nonessential amino acids (Corning, 25–025-Cl), penicillin-streptomycin solution (Corning, 30–002-Cl), 1 mmol/L sodium pyruvate (Corning, 25–000-Cl), and 10% fetal bovine serum (volume fraction; Sigma-Aldrich, F2442). Cells were cultured to 80% confluency at which point the growth media was removed and the cells were rinsed with 1× PBS. The PBS was removed and replaced with serum-free media and the cells were incubated for 18 h. Following serum starvation, the media was removed from the cells and replaced with fresh serum-free media containing 100 ng/ml human epidermal growth factor (Cell Signaling Technology, #8916) and 100 ng/ml human insulin-like growth factor 1 (Cell Signaling Technology, #8917) and cells were incubated for 10 min at 37 °C. Following treatment, the media were removed, the cells rinsed with 1× PBS, trypsinized, and centrifuged at 310 *g*_n_ for 5 min. The cell pellet was rinsed again with 1× PBS and centrifuged at 310 *g*_n_ for 5 min. The final PBS wash was removed and the resulting pellet was frozen on dry ice ethanol and stored at −80 °C.

### Sample Preparation Prior to Distribution

Cell pellets (∼4 × 10^8^ cells) were resuspended in 21 ml of lysis buffer [5% SDS (gravimetric fraction); 50 mmol/L ammonium bicarbonate; protease and phosphatase inhibitor). The solution was incubated on a nutator at 4 °C for 30 min. In total, 143 mg of protein lysate was recovered. For cysteine reduction, 788 μl of 50 mmol/L (20 mM) DTT was added, and the lysate was incubated at 95 °C for 10 min and cooled down to room temperature. For cysteine alkylation, 788 μl of 100 mmol/L (20 mM) iodoacetamide was added and incubated in the dark for 30 min at room temperature. The sample was centrifuged for 10 min at 13,000 *g*_n_ in a tabletop centrifuge. The supernatant was transferred to a new tube and 2106 μl of 12% phosphoric acid (volume fraction) was added (final volume fraction 1.2%) to acidify. The entire lysate was split into four 50 ml tubes and 34.75 ml of S-Trap buffer was added to each tube. The samples were mixed by vortexing and incubated at room temperature for 5 min. The samples were distributed into 28 S-Trap Midi columns. The columns were spun at 4000 *g*_n_ for 30 s until the sample passed through the S-trap column. The sample loading procedure was repeated two more times. The captured proteins were washed by adding 3 ml of S-trap buffer by centrifuging for 30 s at 4000 *g*_n_. The wash step was repeated three more times. A total of 350 μl of digestion solution (6200 μl of 100 mmol/L tetraethylammonium bromide (TEAB) added to 3600 μl of 2 mg/ml trypsin, or 0.73 μg/μl trypsin in 63 mM TEAB) was added to the S-traps and centrifuged at 200 *g*_n_ for 1 min. The S-traps were incubated at 47 °C overnight for trypsin digestion. After digestion, 500 μl of 100 mmol/L TEAB was added to each column and centrifuged at 4000 *g*_n_ for 1 min (first elution). We added 500 μl of 0.2% formic acid (volume fraction) to each column and centrifuged at 4000 *g*_n_ for 1 min (second elution). Five hundred microliters of 50% acetonitrile (volume fraction in water), 0.2% formic acid (volume fraction in water) was added and centrifuged at 4000 *g*_n_ for 1 min (third elution). The empty columns were centrifuged again at 4000 *g*_n_ for 1 min. All the elutions were combined and dried down with a speed vacuum concentrator. The resulting peptides were further purified with a Waters C18 Sep-Pak (35 cubic centimeters, 10 g) according to the manufacturer’s protocol. After resuspending the purified HeLa peptides, the peptide amount was measured as 83.9 mg.

### Sample Distribution

HeLa samples were divided into 1 mg aliquots spiked with 2 pmol of the heavy SIL peptide mixture and then lyophilized in Eppendorf tubes. Tubes were mailed at room temperature to five independent labs for phosphopeptide enrichment and MS/MS analysis.

### Phosphopeptide Enrichment

Phosphopeptides were enriched independently at each lab site using the PTMScan Phospho-Enrichment IMAC Fe-NTA Magnetic Beads (Cell Signaling Technology, Inc., #20432) according to the manufacturer’s recommended protocol. Briefly, aliquots of 20 μl 25% IMAC bead slurry (5 μl packed beads added to 15 μl water) were transferred to two 1.7 ml microcentrifuge tubes. Each aliquot of beads was washed three times with 1 ml IMAC wash buffer [0.1% TFA; volume fraction), 80% acetonitrile (volume fraction in water)]. The 1 mg sample of dried HeLa peptides was resuspended in 1 ml IMAC loading buffer [0.1% TFA (volume fraction), 85% acetonitrile (volume fraction in water)] and 500 μl was transferred to each tube of IMAC beads. Next, 500 μl IMAC loading buffer was added to each tube for 1 ml total volume. Beads were rotated for 30 min at room temperature, the supernatant was removed, and beads were washed three times with 1 ml IMAC wash buffer. Phosphopeptides were eluted from beads two times in 50 μl IMAC elution buffer [50% acetonitrile (volume fraction), 2.5% ammonia (volume fraction)] and acidified with 20% TFA (volume fraction). Eluted phosphopeptides from both tubes were combined, dried in a Speed-Vac, and cleaned up using a single STAGE tip.

### Liquid Chromatography

After following this phosphopeptide enrichment protocol, LC-based MS/MS measurements were performed on different platforms at five distinct lab sites using similar measurement settings.

#### Lab Site A Setup

The resulting phosphopeptide mixture was analyzed using an UltiMate 3000 Nano LC coupled to a Fusion Lumos mass spectrometer (Thermo Fisher Scientific). The sample was loaded onto a PepMap 100 C18 trap column (75 μm id × 2 cm length; 3 μm, 100 Å, C18 resin; Thermo Fisher Scientific) at 3 μl/min for 10 min with 2% acetonitrile (volume fraction) and 0.05% TFA (volume fraction) followed by separation on an Acclaim PepMap RSLC 2 μm C18 column (75 μm id × 25 cm length; Thermo Fisher Scientific) at 40 °C. After loading, peptides were separated along a 120 min two-step gradient of 5% to 27.5% mobile phase B (80% acetonitrile and 0.08% formic acid) over 105 min followed by a ramp to 40% mobile phase B over 15 min. Lastly, the gradient was ramped to 95% mobile phase B over 10 min, and held at 95% mobile phase B for 10 min before returning to 5% mobile phase B, all at a flow rate of 300 nl/min. Mobile phase A is 0.1% formic acid (volume fraction) in water. Data were acquired on the mass spectrometer from 10 min to 150 min.

#### Lab Site B Setup

The resulting phosphopeptide mixture was analyzed using an Easy-nLC 1200 system (Thermo Fisher Scientific) coupled to a Thermo Scientific Q Exactive HF mass spectrometer (Thermo Fisher Scientific). The sample was loaded onto an EASY-Spray ES902 column (75 μm id × 25 cm length; 2 μm, 100 Å, C18 resin; Thermo Fisher Scientific) with 100% mobile phase A (0.1% formic acid (volume fraction) in LC-MS grade water). After loading, peptides were separated along a 120 min two-step gradient of 3% to 26% mobile phase B (85% acetonitrile, 0.1% formic acid; volume fraction) over 90 min followed by a ramp to 40% mobile phase B over 30 min. Lastly, the gradient was ramped to 100% mobile phase B over 1 min, and held at 100% mobile phase B for 3 min before returning to 95% mobile phase A, all at a flow rate of 300 nl/min. Data were acquired on the mass spectrometer throughout the gradient.

#### Lab Site C Setup

The resulting phosphopeptide mixture was analyzed using an Easy-nLC I coupled to a Q Exactive mass spectrometer (Thermo Fisher Scientific). The sample was loaded onto a house-packed analytical column (100 μm id × 22 cm length; packed with 3 μm, 120 Å, C18 resin, Bischoff Chromatography) at 28,000 kPa for 20 min with 100% mobile phase A [2.9% acetonitrile (volume fraction) and 0.12% formic acid (volume fraction)]. After loading, peptides were separated along a 125 min two-step gradient of 5% to 30% mobile phase B (100% acetonitrile, 0.15% formic acid; volume fraction) over 120 min followed by a ramp to 40% mobile phase B over 5 min. Lastly, the gradient was ramped to 98% mobile phase B over 3 min, and held at 98% mobile phase B for 2 min before returning to 100% mobile phase A, all at a flow rate of 300 nl/min. Data were acquired on the mass spectrometer throughout the gradient.

#### Lab Site D Setup

The resulting phosphopeptide mixture was analyzed using an Easy-nLC 1000 coupled to a Fusion mass spectrometer (Thermo Fisher Scientific). The sample was loaded onto a PepMap 100 C18 trap column (75 μm id × 2 cm length; 3 μm, 100 Å, C18 resin; Thermo Fisher Scientific) at 2 μl/min for 15 min with 0.10% formic acid (volume fraction) followed by separation on an Acclaim PepMap RSLC 2 μm C18 EasySpray column (75 μm id × 25 cm length; Thermo Fisher Scientific) with temperature set at 45 °C. After loading, peptides were separated along a 90 min multistep analytical run with mobile phase A being 0.1% formic acid (volume fraction) and mobile phase B being acetonitrile with 0.1% formic acid (volume fraction). The gradient started with 4% B and progressed to 9% B at 28 min, 15% B at 56 min, 24% B at 74 min, 35% B at 79 min, and 95% B at 80 min through the end of the run. The flow rate was maintained at 200 nl/min for the entire analysis and data were acquired for the entire run.

#### Lab Site E Setup

Digested peptides were reconstituted in 2% acetonitrile (volume fraction), 0.1% TFA (volume fraction) and analyzed on a Thermo Scientific Fusion Lumos Orbitrap Mass Spectrometer in conjunction with an UltiMate 3000 RSLCnano UHPLC and EASY-Spray source operating in positive ionization mode. Peptides were loaded on a Thermo Scientific Acclaim PepMap 100 C18 reversed-phase pre-column (DX164199, 100 μm x 20 mm, 100 Å, 5 μm) at 5 μl/min for 6 min before being separated using an EASY-Spray C18 reversed-phase analytical column (ES802, 75 μm x 250 mm, 100 Å, 2 μm) and eluted with an increasing percentage of acetonitrile (0% to 50%; volume fraction) throughout a 180 min gradient at a flow rate of 200 nl/min and heated to 40 °C. Specifically, peptides were separated along a 114 min gradient of 2% to 5% acetonitrile in 0.5 min, then 5% to 50% acetonitrile over 113.5 min. Next, the gradient was ramped to 99% acetonitrile over 1 min and held at 99% acetonitrile for 4 min before returning to 2% acetonitrile, all at a flow rate of 200 nl/min. Data were acquired for the entire run.

### Mass Spectrometry

GPF-DIA data acquisition was performed individually at each lab site following the protocols described in Pino *et al.* (25) Briefly, each lab acquired eight GPF-DIA acquisitions with 4 m/z DIA spectra at 30,000 resolution and 55 ms maximum ion injection time. Thermo QE and QE-HF instruments (lab sites B, C, and the library generation site) were configured to use an automatic gain control (AGC) target of 1 x 10^6^ ions and a normalized collision energy (NCE) of 27. Thermo Fusion and Fusion Lumos tribrid instruments (lab sites A, D, and E) were configured to acquire higher-energy collisional dissociation MS/MS using the orbitrap detector with an AGC target of 4 x 10^5^ ions and an NCE of 30. For all instruments, windows were configured in a staggered window placement with optimized window boundaries to place window boundaries near “forbidden zones” (46) (*i.e.*, 398.43 m/z to 502.48 m/z, 498.48 m/z to 602.52 m/z, 598.52 m/z to 702.57 m/z, 698.57 m/z to 802.61 m/z, 798.61 m/z to 902.66 m/z, 898.66 m/z to 1002.70 m/z, 998.70 m/z to 1102.75, and 1098.75 m/z to 1202.80 m/z). Orbitrap precursor spectra were acquired and matched to each window range (*i.e.*, 390 m/z to 510 m/z, 490 m/z to 610 m/z, 590 m/z to 710 m/z, 690 m/z to 810 m/z, 790 m/z to 910 m/z, 890 m/z to 1010 m/z, 990 m/z to 1110 m/z, and 1090 m/z to 1210 m/z). Lab instrumentation setups are summarized in Table 1.

**Table 1:** Summary of HPLC types, LC columns, LC gradients, and mass spectrometers used at each site

Lab site	HPLC	LC column	LC gradient	MS
A	Ultimate 3000	PepMap RSLC 2 μm C18 column (75 μm id × 25 cm length)	300 nl/min 120 min two-step separation	Fusion Lumos Orbitrap
B	Easy-nLC 1200	EASY-Spray ES902 column (75 μm id × 25 cm length; 2 μm, 100 Å, C18 resin)	300 nl/min 120 min two-step separation	Q Exactive HF
C	Easy-nLC I	house-packed analytical column (100 μm id × 22 cm length; packed with 3 μm, 120 Å, C18 resin)	300 nl/min 125 min two-step separation	Q Exactive
D	Easy-nLC 1000	Acclaim PepMap RSLC 2 μm C18 EasySpray column (75 μm id × 25 cm length)	200 nl/min 90 min multi-step separation	Fusion Orbitrap
E	Ultimate 3000	EASY-Spray C18 reversed-phase analytical column (ES802, 75 μm x 250 mm, 100 Å, 2 μm)	200 nl/min 114 min two-step separation	Fusion Lumos Orbitrap

### Phosphopeptide Library Generation

The SIL phosphopeptides were also measured alone in water without an additional background to generate a library. Phosphopeptides were separated with a Waters NanoAcquity UPLC and emitted into a Thermo Q-Exactive HF mass spectrometer. For each injection, a 90 min separation was performed using a pulled tip 75 μm inner diameter fused silica column, which was created packed with 3 μm ReproSil-Pur C18 beads (Dr Maisch) to 300 mm and a similar 150 μm inner diameter trap column packed to 25 mm. Nine GPF-DIA experiments covering 400 m/z to 1300 m/z in 100 m/z width injections were performed using 500 fmol total phosphopeptides to test the presence of each peptide and identify the best-responding charge state for each peptide. Each injection was acquired with 51 DIA spectra (4 m/z precursor isolation windows at 30,000 resolution, AGC target 1e6, maximum inject time 55 ms, 27 NCE) using the same staggered window pattern as the standard analysis. Two precursor spectra, a wide spectrum (400 m/z to 1600 m/z at 60,000 resolution) and a narrow spectrum matching the range (*i.e.* 390 m/z to 510 m/z, 490 m/z to 610 m/z, 590 m/z to 710 m/z, 690 m/z to 810 m/z, 790 m/z to 910 m/z, 890 m/z to 1010 m/z, 990 m/z to 1110 m/z, 1090 m/z to 1210 m/z, and 1190 m/z to 1310 m/z) were interspersed every 25 MS/MS spectra configured with an AGC target of 3e6 and a maximum injection time of 100 ms.

The resulting GPF-DIA datasets were demultiplexed using Proteowizard (version 3.0.18299) using the settings “--simAsSpectra --zlib --64 --mzML --filter “peakPicking true 1-” --filter “demultiplex optimization=overlap_only” ∗.raw” and analyzed using Skyline-daily (version 4.1.1.11903). Peptide chromatograms were manually validated based on fragmentation similarity, retention time alignment, and mass accuracy *versus* entries in the Phosphopedia library (12). Confidently measured peptides were exported as a BLIB spectrum library file and iRTDB retention time calculator file. While nine GPF-DIA fractions were collected for library generation, we observed that few if any peptides were best observed in the 1200 m/z to 1300 m/z fraction. As a result, this fraction was not measured by the individual lab sites.

### Targeted Data Analysis

GPF-DIA data were demultiplexed using Proteowizard using the same settings as above and analyzed using Skyline (21.2.0.425). Skyline was configured to extract precursor, b, b++, y, and y++ ions from ion three to the last ion. Up to 12 library ions were chosen with an ion match tolerance of 0.5 m/z. The method match tolerance was configured to 0.055 m/z and a 10 ppm tolerance was used to extract ions from the mzML files across all matching spectra. Using the phosphopeptide library and retention time calculator above, each peptide was manually integrated to remove fragment ions with interference as well as peptides without sufficient signal in the heavy channel. All manual integrations for all lab sites (A-E) were completed by a single individual at the library generation lab site to maintain consistency. Total MS1 and MS2 peak areas were exported for each peptide in each lab site across all GPF injections. Total peak areas were summed across charge states for reporting.

## Results and Discussion

### Peptide Selection and Characterization

General purpose phosphopeptide standards are typically designed with several factors in mind. Along with other groups, over the last two decades, the Proteomics Standards Research Group (sPRG) has generated several phosphopeptide standards (47) that have primarily focused on workflow evaluation. These types of standards enable researchers to test sample preparation workflows and instrument configurations on well-characterized sets of phosphopeptides with a broad range of physicochemical properties.

Phosphopeptide enrichment methods using metal-ion affinity or antibodies are manually intensive tasks where significant errors can be introduced. While robotics can help standardize phosphopeptide enrichment workflows (26), minor changes can have large effects on efficiency, which can be additionally problematic if those changes affect a subpopulation of peptides, for example, only doubly and triply phosphorylated peptides (48). As such, multiple standards now exist to evaluate and normalize for enrichment quality on a sample-by-sample basis.

**Fig. 1:**
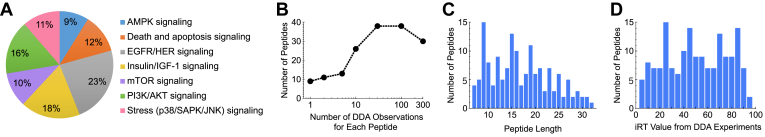
**Peptide properties for the multi-pathway phosphopeptide standard.***A*, pie chart showing the relative breakdown of selected phosphopeptides across signaling pathways. Histograms showing (*B*) the number of observations for each peptide in the Phosphopedia database (log_10_ scale), (*C*) the distribution of peptide lengths, and (*D*) the distribution of estimated iRT values. iRT, indexed retention time.

In this work, we sought to generate a phosphoproteomics standard to speed up assay development for common biological pathways in humans. Many cellular signaling pathways are regulated by the same kinases that phosphorylate “hub and spoke” phosphosites, and here we exploit that to generate a concise collection of sites. Working from sites observed in the Phosphopedia online resource (12), we initially selected 179 human phosphopeptides containing sites with known biological effects in seven different signaling pathways: AMPK signaling, death and apoptosis signaling, ErbB signaling, insulin/IGF-1 signaling, mTOR signaling, PI3K/AKT signaling, and stress (p38/SAPK/JNK) signaling (Fig. 1*A*).

Of the 179 targets initially selected by bioinformatic analysis, we performed the synthesis of 150 phosphopeptides from 89 proteins, covering 96 serine, 28 threonine, and 36 tyrosine sites of phosphorylation. Beyond desalting, these peptides were unpurified “crude” SIL peptides and in some cases were of poor purity as validated by MS1 signal. While some phosphopeptides have been observed hundreds of times in Phosphopedia, others have only been reported in that library a single time (Fig. 1*B*). If possible, for each site we selected the most common (by the number of observations) singly phosphorylated peptide (143 total), choosing doubly (6 total) and triply (1 total) phosphorylated peptides only when necessary. These 150 peptides were additionally selected to span a wide range in amino acid length (Fig. 1*C*) and relative indexed retention time estimates to make their observation easier to schedule in a complex background (Fig. 1*D*).

We analyzed these 150 heavy-labeled phosphopeptides using a nine-injection GPF-DIA scheme measuring signals between 400 m/z and 1300 m/z in 100 m/z width injections. These measurements were made using staggered 4 m/z windows, effectively achieving 2 m/z precursor isolation after staggered demultiplexing using Proteowizard (49). We integrated these peptides using Skyline (50) to check for appropriate abundance, to identify peptides that potentially resolved poorly chromatographically, and to build a DIA-specific phosphopeptide library. The library-generation lab was kept separate from all five test lab sites in order to more accurately mimic library usage in other labs outside of our working group.

Of the 150 SIL peptides, 128 produced a high signal and resolved well, and 10 produced a low signal but resolved well. From this, we constructed a DIA-specific spectrum library containing 138 peptides, some of which were represented by multiple charge states (233 total spectra). Of the remaining peptides, five either resolved poorly with broad peaks >1 min wide or produced signals that were difficult to distinguish from noise and seven did not produce any observable signals within the 400 m/z to 1300 m/z range. A similar analysis of these peptides in a HeLa background indicated that 122 of 138 peptides continued to resolve well in a background proteome. Of the 16 remaining peptides, eight resolved poorly while eight produced signals indistinguishable from the background.

### Multilab Validation and Analysis

**Fig. 2:**
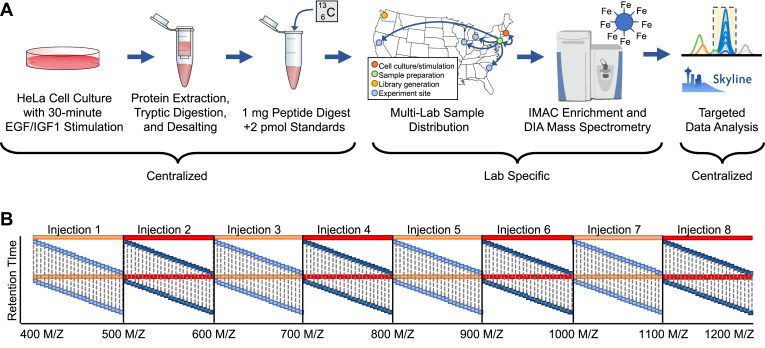
**Experimental design.***A*, a schematic showing key steps in our experimental approach to assess the lab-to-lab variability of the multipathway phosphopeptide standard. Key steps, such as cell culture, digestion, mixing, and data analysis were controlled, while variability from IMAC enrichment and mass spectrometry instrumentation was isolated in each lab site. *B*, all lab sites were instructed to use the same eight-injection GPF-DIA method to ensure consistency between lab sites. In this approach, eight injections of the same sample were made, spanning 100 m/z ranges. Each injection was performed using staggered windowing to achieve 2 m/z precursor isolation (targeted PRM equivalent isolation). DIA, data-independent acquisition; GPF, gas-phase fractionation; IMAC, immobilized metal ion affinity chromatography; PRM, parallel reaction monitoring.

We validated our multipathway phosphopeptide standard by monitoring endogenous peptides in a HeLa background. To this end, we stimulated HeLa cells for 10 min using both EGF and IGF-1 after starvation for 18 h to synchronize cells. While there is significant crosstalk between phosphorylation signaling pathways, IGF-1 generally stimulates the PI3K/AKT/mTOR pathway, while EGF stimulates the MAPK/ERK pathway. This mixed pathway stimulation allowed us to test a significant fraction of sites covered by the multipathway phosphopeptide standard in a single experiment. As described in Figure 2*A*, we centrally lysed, reduced alkylated, and digested the resulting cell pellets. We then distributed approximately 1 mg of the resulting peptide samples, adding 2 pmol of the heavy phosphopeptide standards, to five independent labs across the country. At each lab site, we enriched for phosphopeptides using a standardized IMAC protocol using Fe-NTA magnetic beads in the PTMScan IMAC kit from Cell Signaling Technologies. Each lab analyzed these samples using a common LC-MS/MS workflow based on GPF-DIA mass spectrometry methods, where each lab used independent LC-MS/MS instrumentation (Fig. 2*B*). This method was based on the same approach used for library generation and allowed us to test the lab-to-lab variability in phosphopeptide enrichment and mass spectrometry working from a standardized sample and standardized methods. While this method required more injections than a typical targeted PRM experiment, it allowed us to acquire PRM-quality data while sidestepping a potential source of lab-to-lab variability with retention time scheduling.

**Fig. 3:**
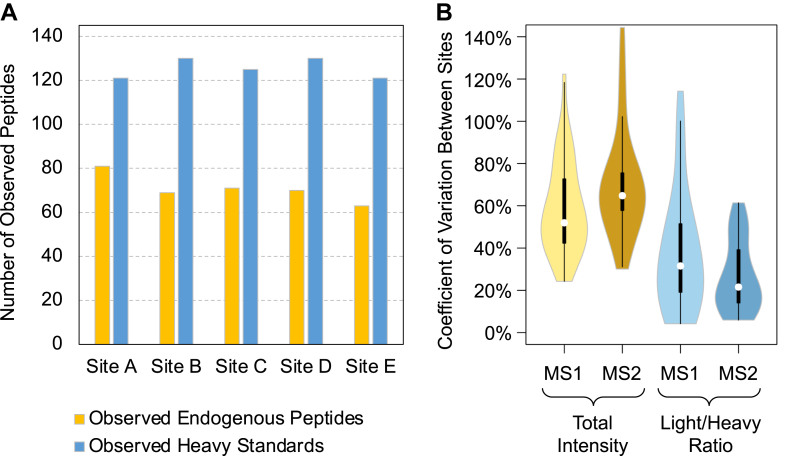
**Measurement consistency across labs.***A*, the total number of observed heavy and endogenous peptides at each lab site. For each peptide, the heavy form must be confidently detected (correct retention time, fragmentation, and mass accuracy) for the corresponding endogenous peptide to be considered “observed”, ensuring a light/heavy ratio. Similarly, endogenous peptides were only “observed” if they had light/heavy ratios >1/100 to protect against integrating noise. *B*, a violin plot showing the CV between measurements across labs for the top 30 peptides (first two orders of magnitude in ratio) using either total intensity or the light/heavy ratio for both MS1 and MS2 data. *Black boxes* indicate the interquartile range, while the *white points* indicate the median CV.

We found that the overall number of observed heavy and endogenous peptides was surprisingly similar between lab sites, despite the measurements being performed with different platforms spanning several generations (51) of orbitrap-based mass spectrometers (Fig. 3*A*). The number of heavy peptides ranged from 121 to 130 (average = 125), while the number of light peptides ranged from 63 to 81 (average 71). We found that heavy peptide measurements were quite consistent between labs, where 134 of 135 total measured heavy peptides (99%) were observed by at least two of the labs, and 127 (94%) were observed by at least four of the labs (supplemental Fig. S1*A*). In contrast, only 80 of 104 total measured endogenous peptides (77%) were observed by at least two of the labs (supplemental Fig. S1*B*).

By maximizing ion injection times for a sufficient length on each MS/MS window, (in this study, 50 msec to 60 msec per window), the number of detections did not scale with instrument age or model, where the oldest instrument (a Q-Exactive Classic at lab site C) performed similarly to the newest instrument (Fusion Lumos at lab sites A and E). This finding underlines the reproducibility of GPF-DIA quantification with orbitrap instruments as an alternative data acquisition approach to achieve PRM-like precision. Based on this result, we believe that especially with lower complexity samples like those produced by phosphopeptide enrichments, GPF-DIA measurements are more limited by obtaining sufficient numbers of ions (*e.g.*, ion flux) than by instrument sensitivity or resolution. Thus, maximizing ion injection time on a trapping detector such as the Orbitrap mass analyzer can lessen the limitation of older instrumentation. Future work is needed to further investigate this result.

In this experiment, we stimulated both the IGF-1 and EGF pathways to activate the MAPK and PI3K/AKT/mTOR pathways in HeLa. As a result, we expect some but not all of the phosphopeptides to be expressed endogenously. That said, we expect that the relative light/heavy ratios should be consistent across lab sites. With centralized data analysis, any lab-to-lab variation observed in these ratios should be due to differences in the LC-MS method, instrumentation, or phosphopeptide recovery during the lab site-specific IMAC enrichment step. We found that the MS2 quantification ratios observed at each lab site were very precise across two orders of magnitude with a median CV of 21.6% (Fig. 3*B*), and reasonably well across three orders of magnitude (median CV = 32.8%). We found that light/heavy ratios were highly significantly more consistent between labs than MS1 (median CV = 51.9%, *p*-value = 2.0e-8) and MS2 total intensities (median CV = 64.8%, *p*-value = 9.6e-11), even though (a) all measurements were performed on Thermo Orbitraps, (b) total intensity datasets were median normalized to remove global bias, and (c) peak intensities were estimated in the same units (ions per second). Peptide abundance variation reflects not just global instrumentation bias but also variability introduced through sample preparation. Since all five sites used independent phosphopeptide enrichment, peptide-to-peptide differences in enrichment made it difficult to calibrate between sites (or even between sample preparations) without a common reference standard (52). Interestingly, MS2-level intensities, which are typically reported in PRM and DIA experiments, are the least consistent between labs most likely due to lab-specific differences in transition selection as part of data analysis.

Both MS1- and MS2-level light/heavy ratios show a high degree of consistency between labs. Since the heavy phosphopeptide standard was added before the enrichment step, lab-to-lab variability in phosphopeptide enrichment affects the sample and the standard equally. This result suggests that heavy phosphopeptide standards can act as a peptide-by-peptide correction factor between sample preparations and even between labs, ensuring consistent quantification with low CVs. As such, we believe that standards like the one we present here could enable quantitative experiments with large scopes that extend beyond what can be performed in a single laboratory.

**Fig. 4:**
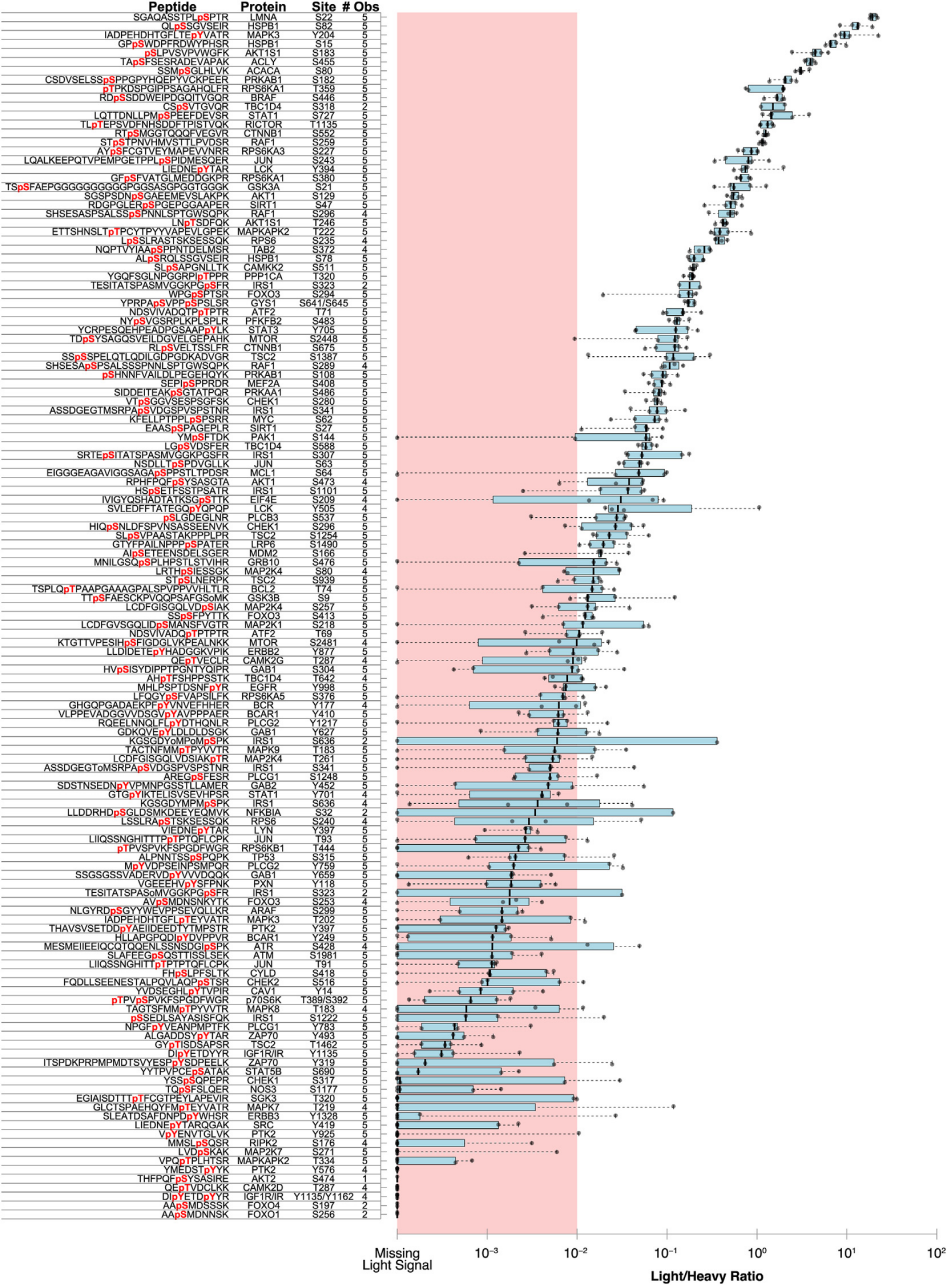
**Peptide quantification accuracy across sites.***Box plots* show the median and estimated quartiles of log_10_ normalized light/heavy ratios for confidently observed heavy peptides at each lab site. *Whiskers* indicate the full range of values, while *gray dots* indicate the actual ratios for each site. Individual light intensity values in the *pink* shaded region (with below 1/100 light/heavy ratio) are considered low confidence and marked as “unobserved” in Figure 3. In addition to the sequence, protein, and site, the number of lab sites that confidently observed each heavy peptide (of five total lab sites) is also indicated. Peptides are sorted by the median light/heavy ratio.

In general, light/heavy ratio precision between labs scaled with fold change (Fig. 4). Peptides with median fold changes estimated above 1:100 light/heavy were typically measured consistently at every lab site (94% of measurements had both light and heavy peptides), while peptides below that were based on missing (heavy absent) or 0 (light absent) integration values 39% of the time. All quantitative data are detailed in supplemental Table S2. In this experiment, we performed simultaneous IGF-1 and EGF stimulation in order to increase the possibility of observing endogenous signaling in these pathways and did not perform a stimulated/unstimulated assay. As a result, in this experiment the light/heavy ratios do not indicate which sites were stimulated; the ratios only indicate that the sites are observable in the background of HeLa phosphorylation relative to our heavy spike-in phosphopeptides. That said, many of the intensely observed sites are associated with either the AKT1 pathway (*e.g.*, HSPB1 S82) or the MAPK pathway (*e.g.*, HSPB1 S15).

**Fig. 5:**
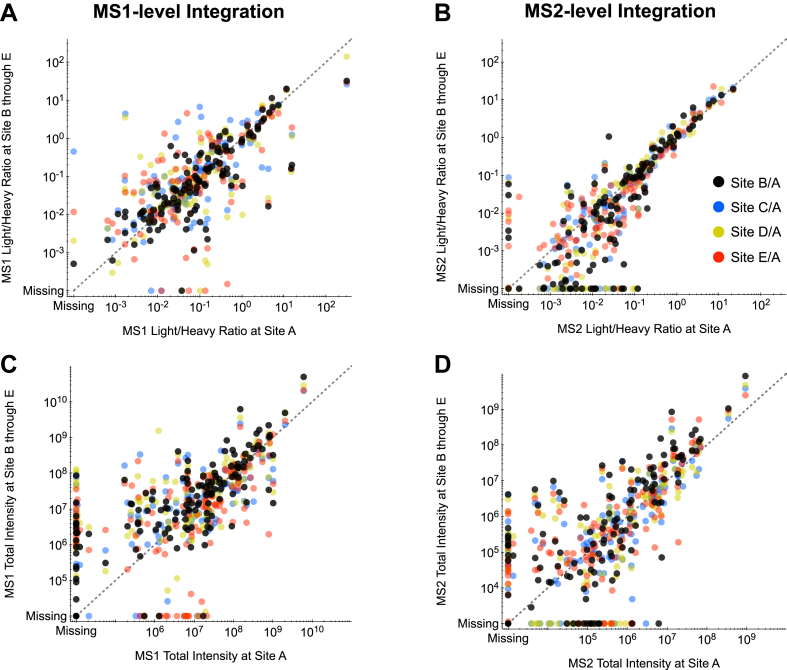
**Comparison of MS1- and MS2-level quantification.** Phosphopeptide MS1-level (*A*) and MS2-level light/heavy ratios (*B*), as well as MS1-level (*C*) and MS2-level total light intensities (*D*) for lab sites B, C, D, and E relative to A. *Dashed lines* are shown to indicate matching 1:1 agreement between lab-specific measurements. Peptide ratio of ratios that fit closer to the *dashed line* show lower variability between labs. All axes were selected to show approximately six orders of magnitude.

In addition to MS2 quantification, the GPF-DIA method we used here also collected consistent MS1 spectra that could be used for quantification. While MS1 signals are typically not used for quantification in DIA experiments, precursor integration is commonly used to quantify peptides in DDA experiments. Using the same retention time boundaries with Skyline, we integrated both MS1 and MS2 signals for each detected peptide and computed light/heavy ratios. In general, both integration approaches agreed. Using lab site A as a relative benchmark, we found that MS1-level light/heavy ratios showed higher variability between lab sites (Fig. 5*A*) than MS2-level integrations (Fig. 5*B*), reflecting the somewhat higher MS1-level CV shown in Figure 3*B* (median CV = 31.5%). For this comparison, lab site A was chosen as a benchmark because it detected the highest number of endogenous peptides. While it may be possible to use MS1-level data to improve MS2-based quantifications (53), this result shows the significant limitation of relying on MS1-level data alone for quantification, even in lower complexity samples such as phosphopeptide enrichments. This is in part due to the fact that some fragment ions with interference can be removed through transition refinement without negatively affecting the quantitative results, while precursor ions typically cannot be removed in a way that still maintains at least three independent quantitative measurements of the peptide. Total intensities measured in each lab at the MS1-level (Fig. 5*C*) and MS2-level (Fig. 5*D*) show increased scatter off the ideal 1:1 line, even after accounting for bias with median normalization between datasets.

**Fig. 6:**
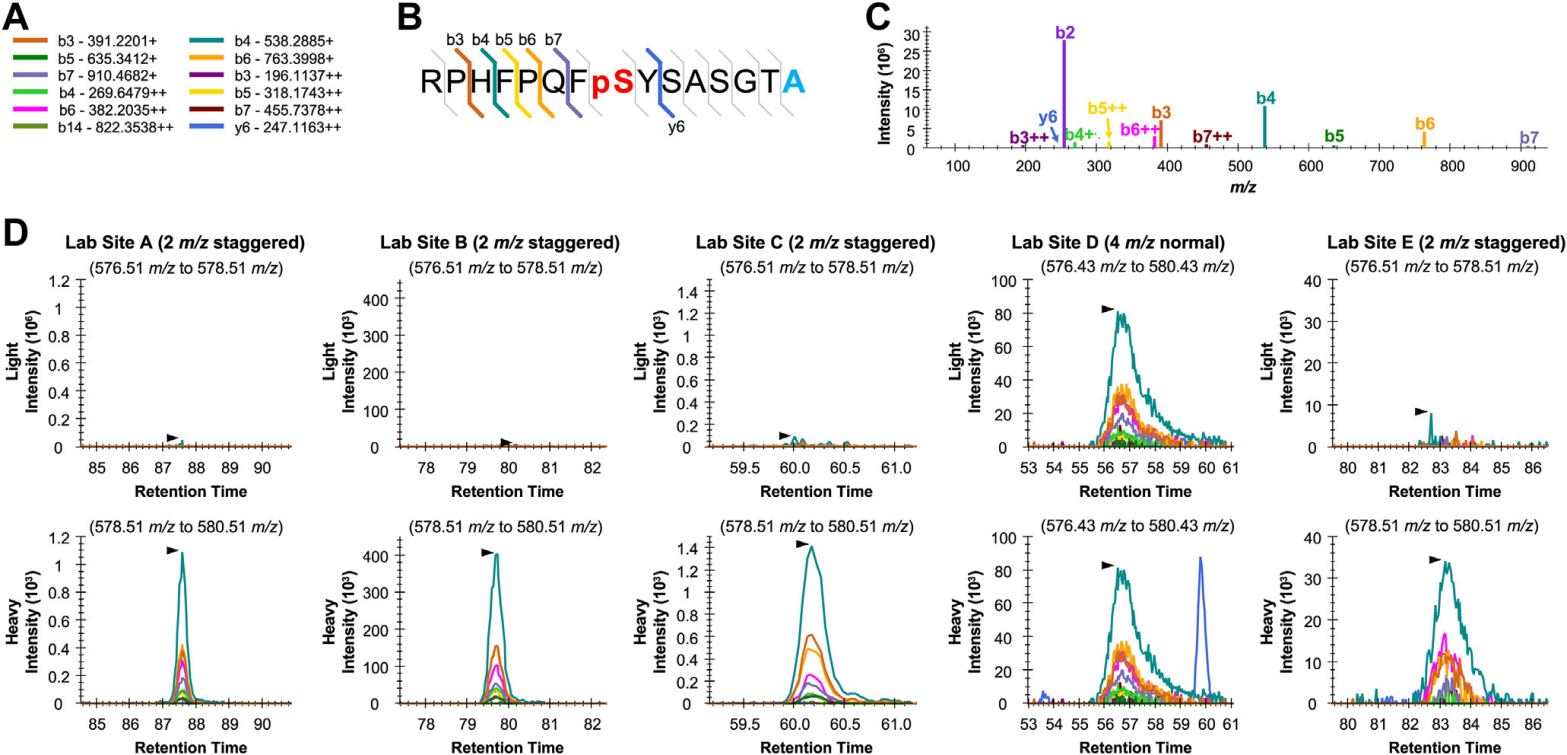
**Challenges of measuring endogenous AKT1 S473 with wide DIA windows.** The peptide RPHFPQFpSYSASGTA produces several fragment ions (*A*), producing a long consecutive b-ion ladder from b3 to b7 (*B*), but few y-type ions, as demonstrated by the library entry spectrum (*C*). The lack of fragment ions containing the heavy-labeled C-terminal alanine residue (*light blue*) means that these ions must be quantified either from precursors or by separating the light (578.256 m/z) and heavy (579.592 m/z) forms into different precursor isolation windows. *D*, staggering 4 m/z windows to achieve 2 m/z isolation, as performed by lab sites A, B, C, and E can separate light and heavy integrations. However, normal 4 m/z windows, as performed by lab site D, cofragment light and heavy peptides such that their b-ion signals cannot be separated. DIA, data-independent acquisition.

The change in mass caused by the heavy amino acids used in the phosphopeptide mixture range from +4 Da (A) to +10 Da (R). These heavy amino acids were intentionally placed on the peptide C terminus making it easier to differentiate light and heavy peptides using the C-terminal y-ion series. However, this has important implications for how the mixture can be used because both light and heavy peptides produce the same N-terminal b-ion series ions and care must be taken when monitoring these ions. For example, the peptide representing AKT1 S473 is RPHFPQFpSYSASGTA with one heavy alanine residue. In our study, all five lab sites measured this heavy peptide as +3H, which produces a strong b-ion ladder but few if any y-ions (Fig. 6, *A*–*C*). The S473 site is phosphorylated by mTOR downstream of IGF1R and is one of the most frequently observed peptides in Phosphopedia (12), but the endogenous form is not observed in this experiment. However, the interpretation with DIA methods is complicated by the small m/z difference between the light and heavy forms (1.336 m/z) such that both forms of the peptide can fall in the same precursor isolation window. While the 4 m/z staggered window method used by lab sites A, B, C, and E can be demultiplexed into two 2 m/z windows that can separate the light and heavy form, lab site D used 4 m/z normal (unstaggered) windows where the light and heavy peptides are cofragmented in the same window such that the heavy signal can be misinterpreted as coming from the light peptide (Fig. 6*D*). This example underlines the challenges arising from interpreting SIL peptides collected with windows wider than 2 m/z, either from DIA (54) or wide-window DDA (55) measurements.

### Challenges and Opportunities With Measuring Heavy Phosphopeptide Standards

**Fig. 7:**
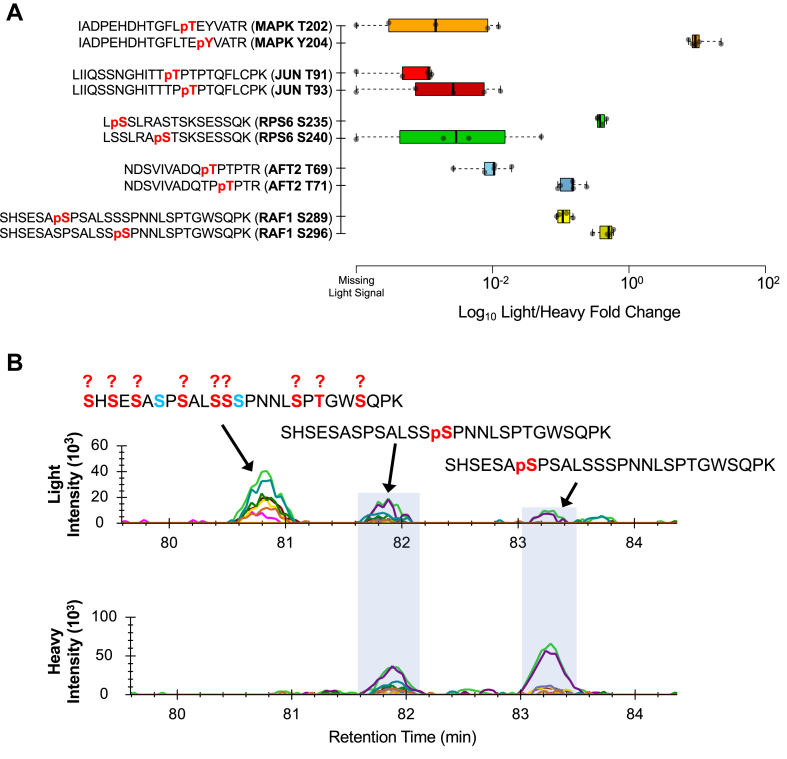
**Quantifying phosphopeptide positional isomers.** Relative light/heavy ratios for five positional isomer pairs in the phosphopeptide mixture (*A*). Fragment ion chromatograms for positional isomers in the RAF1 peptide SHSESASPSALSSSPNNLSPTGWSQPK (*B*). Here, S289 and S296 (shaded in *gray boxes*) are indicated by heavy fragment ion signals, while a third unknown positional isomer of this peptide does not time align with either site.

Proteins are frequently phosphorylated at neighboring sites (56, 57) where each phosphosite can have different biological functions (58) and must be measured independently to fully characterize the upstream or downstream biology. Peptide-to-phosphosite assignment is typically performed using site localization algorithms (59, 60, 61, 62, 63, 64, 65), but it can also be performed using heavy standards. The phosphopeptide mixture in this study contains five sets of positional isomers and several peptides in both singly and doubly phosphorylated states. For example, MAPK3 (also known as ERK1) is activated by phosphorylation at both T202 and Y204 by MEK1 in the MAPK signal transduction pathway (66). After stimulating with EGF for only 10 min, we were able to consistently differentially measure T202 and Y204 across all five lab sites (Fig. 7*A*) where Y204 monophosphorylation was clearly observed while the T202 monophosphorylated species were not. Although the fragment intensity signals are not directly comparable, this result confirms the precise ordering of phosphorylation events in the ERK1 activation loop where tyrosine phosphorylation precedes threonine phosphorylation (67). Similarly, the JUN T91/T93, AFT2 T69/T71, and RAF1 S289/S296 sites are all thought to be ERK substrates and the inclusion of these positional isomers could act as detailed temporal map of ERK activity. Future work is needed to explore the utility of monitoring these peptides as a panel.

Additionally, the phosphopeptide mixture can help assign positional isomers in challenging peptides. For example, RAF1 is putatively phosphorylated at S289 and S296 by MEK1 (68). The peptide containing these phosphosites has ten serines and one threonine that could be potentially phosphorylated, making consistent site localization based on the observation of site-determining fragment ions nearly impossible. In our study, all of the lab sites observed at least three positional isomers, where the most abundant form with the highest number of assigned fragment ions consistently eluted earlier than either S289 or S296 (Fig. 7*B*).

The combinatorial nature of phosphorylation acceptor sites in peptides underlines another consideration. A limitation of phosphopeptide standards is that they cannot comprehensively contain every potential positional isomer and that some biologically active phosphosites will not be included. For example, this standard contains singly phosphorylated DIpYETDYYR and doubly phosphorylated DIpYETDpYYR, which map to Y1161 and Y1165 in IGF1R, the two most commonly observed sites in PhosphoSitePlus (13). However, the standard is missing other potential positional isomers of this peptide including T1163 and Y1166, which have both been observed in over 50 publications. One advantage of DIA is that it enables the potential to look for unexpected positional isomers at alternate retention times or m/z ranges (28). Resolving these forms from gas-phase rearrangements (69) without SIL standards remains an open challenge.

## Conclusions

The proteomics standards research group of the Association for Biomolecular Resource Facilities developed and validated a multipurpose, SIL phosphopeptide mixture of biologically meaningful phosphosites. This standard will enable researchers to rapidly prototype mass spectrometry assays for key phosphoproteins. In a single run, researchers can monitor dozens of sites with more specificity than Western blots, where both protein isomers with similar sequences and positional isomers with identical sequences can be measured with confidence. The multipathway nature of this standard focusing on measuring hub kinases will have immediate applicability to a wide variety of fields, including cancer, aging, and metabolism. In addition, the standard can be used for quality control, as a phosphopeptide retention time standard, or for technology development with new methods including those outside of mass spectrometry (70). While we demonstrate the utility of the standard using GPF-DIA, the design of this experiment was to mimic PRM-quality MS2 data and DDA-quality MS1 data without needing to schedule peptides or rely on instrument control software to select peptides for measurement.

Over the last 15 years, the sPRG has developed several standards with a wide variety of commercial partners to improve community-wide reproducibility and facilitate proteomics-based team science. The sPRG plays an ongoing role in interfacing between the research community needs and commercial partners. To this end, this multipathway phosphopeptide mixture has been developed into a widely available product by Thermo Fisher Scientific. Some changes exist between that product and the mixture we present here. First, some inconsistent peptides were removed due to stability concerns at −20 °C or synthesis challenges with longer peptides, resulting in a final 131 phosphopeptide standard. These peptides have been purified to >97% followed by peptide content determination by amino acid analysis, which will potentially open the door to a new range of experiments focused on absolute quantification. supplemental Table S3 contains detailed information on the providence of each peptide in the starting assay down to those that were validated in this final list.

Finally, we have demonstrated the tractability of cross-laboratory studies using heavy peptide standards and DIA. Despite wide ranges in mass spectrometry instrumentation and background with phosphopeptide enrichments, we found that all labs could produce reproducible, harmonized measurements of challenging phosphosites as long as results were reported as ratios relative to the standard. This result suggests that when researchers report quantitative ratios to this standard, those results should be directly comparable without any additional data harmonization. Leveraging a widely accessible standard, we believe that this work provides a roadmap for future phosphoproteomics studies that extend beyond the capabilities of any individual laboratory and gives direction to how the proteomics community can grow to empower reproducible science.

## Data Availability

All raw data is publicly available on MassIVE using dataset identifier MSV000090564 (https://doi.org/10.25345/C54J0B266). A data key for the file associations as well as parameters broken out by lab site can be found in supplemental Table S1. All chromatographic data is publicly available for visualization and manual validation at PanoramaWeb using the URL https://panoramaweb.org/Ohio%20State%20University%20-%20Searle%20Lab/sprg2018%20multipathway%20phosphopeptide%20standard/project-begin.view

## Supplemental data

This article contains supplemental data.

## Conflict of interest

B. C. S. is a founder and shareholder in Proteome Software, which operates in the field of proteomics. A. J. N. and J. M. R. are employees of Cell Signaling Technology. A. W. H. and B. P. are employees of Thermo Fisher Scientific.

## Supplementary Data

Supplementary figures Peptide observation consistency across labs.

Table S1 Data key for file associations and parameters.

Table S2 Quantitative data for Figures 3, 4, 5, and 7.

Table S3 Table of 179 starting peptides and their providence.

Table S4 Table of final 138 phosphopeptide targets and associated metadata.

## References

[1] Cohen P. (2002). Protein kinases—the major drug targets of the twenty-first century?. Nat. Rev. Drug Discov..

[2] Ferguson F.M., Gray N.S. (2018). Kinase inhibitors: the road ahead. Nat. Rev. Drug Discov..

[3] Andersson L., Porath J. (1986). Isolation of phosphoproteins by immobilized metal (Fe3+) affinity chromatography. Anal. Biochem..

[4] Stensballe A., Andersen S., Jensen O.N. (2001). Characterization of phosphoproteins from electrophoretic gels by nanoscale Fe(III) affinity chromatography with off-line mass spectrometry analysis. Proteomics.

[5] Pinkse M.W.H., Uitto P.M., Hilhorst M.J., Ooms B., Heck A.J.R. (2004). Selective isolation at the femtomole level of phosphopeptides from proteolytic digests using 2D-NanoLC-ESI-MS/MS and titanium oxide precolumns. Anal. Chem..

[6] Beausoleil S.A., Jedrychowski M., Schwartz D., Elias J.E., Villén J., Li J. (2004). Large-scale characterization of HeLa cell nuclear phosphoproteins. Proc. Natl. Acad. Sci. U. S. A..

[7] Rush J., Moritz A., Lee K.A., Guo A., Goss V.L., Spek E.J. (2005). Immunoaffinity profiling of tyrosine phosphorylation in cancer cells. Nat. Biotechnol..

[8] Zhang Y., Wolf-Yadlin A., Ross P.L., Pappin D.J., Rush J., Lauffenburger D.A. (2005). Time-resolved mass spectrometry of tyrosine phosphorylation sites in the epidermal growth factor receptor signaling network reveals dynamic modules. Mol. Cell Proteomics.

[9] Stokes M.P., Rush J., Macneill J., Ren J.M., Sprott K., Nardone J. (2007). Profiling of UV-induced ATM/ATR signaling pathways. Proc. Natl. Acad. Sci. U. S. A..

[10] Rikova K., Guo A., Zeng Q., Possemato A., Yu J., Haack H. (2007). Global survey of phosphotyrosine signaling identifies oncogenic kinases in lung cancer. Cell.

[11] Stokes M.P., Farnsworth C.L., Moritz A., Silva J.C., Jia X., Lee K.A. (2012). PTMScan direct: identification and quantification of peptides from critical signaling proteins by immunoaffinity enrichment coupled with LC-MS/MS. Mol. Cell Proteomics.

[12] Lawrence R.T., Searle B.C., Llovet A., Villén J. (2016). Plug-and-Play analysis of the human phosphoproteome by targeted high-resolution mass spectrometry. Nat. Methods.

[13] Hornbeck P.V., Kornhauser J.M., Latham V., Murray B., Nandhikonda V., Nord A. (2018). 15 Years of PhosphoSitePlus®: integrating post-translationally modified sites, disease variants and isoforms. Nucl. Acids Res..

[14] Ochoa D., Jarnuczak A.F., Viéitez C., Gehre M., Soucheray M., Mateus A. (2020). The functional landscape of the human phosphoproteome. Nat. Biotechnol..

[15] Stahl D.C., Swiderek K.M., Davis M.T., Lee T.D. (1996). Data-controlled automation of liquid chromatography/tandem mass spectrometry analysis of peptide mixtures. J. Am. Soc. Mass Spectrom..

[16] Bantscheff M., Lemeer S., Savitski M.M., Kuster B. (2012). Quantitative mass spectrometry in proteomics: critical review update from 2007 to the present. Anal. Bioanal. Chem..

[17] Peterson A.C., Russell J.D., Bailey D.J., Westphall M.S., Coon J.J. (2012). Parallel reaction monitoring for high resolution and high mass accuracy quantitative, targeted proteomics. Mol. Cell Proteomics.

[18] Venable J.D., Dong M.-Q., Wohlschlegel J., Dillin A., Yates J.R. (2004). Automated approach for quantitative analysis of complex peptide mixtures from tandem mass spectra. Nat. Methods.

[19] Gillet L.C., Navarro P., Tate S., Röst H., Selevsek N., Reiter L. (2012). Targeted data extraction of the MS/MS spectra generated by data-independent acquisition: a new concept for consistent and accurate proteome analysis. Mol. Cell Proteomics.

[20] Poulos R.C., Hains P.G., Shah R., Lucas N., Xavier D., Manda S.S. (2020). Strategies to enable large-scale proteomics for reproducible research. Nat. Commun..

[21] Collins B.C., Hunter C.L., Liu Y., Schilling B., Rosenberger G., Bader S.L. (2017). Multi-laboratory assessment of reproducibility, qualitative and quantitative performance of SWATH-mass spectrometry. Nat. Commun..

[22] Panchaud A., Scherl A., Shaffer S.A., von Haller P.D., Kulasekara H.D., Miller S.I. (2009). Precursor acquisition independent from ion count: how to dive deeper into the proteomics ocean. Anal. Chem..

[23] Ting Y.S., Egertson J.D., Bollinger J.G., Searle B.C., Payne S.H., Noble W.S. (2017). PECAN: library-free peptide detection for data-independent acquisition tandem mass spectrometry data. Nat. Methods.

[24] Searle B.C., Pino L.K., Egertson J.D., Ting Y.S., Lawrence R.T., MacLean B.X. (2018). Chromatogram libraries improve peptide detection and quantification by data independent acquisition mass spectrometry. Nat. Commun..

[25] Pino L.K., Just S.C., MacCoss M.J., Searle B.C. (2020). Acquiring and analyzing data independent acquisition proteomics experiments without spectrum libraries. Mol. Cell Proteomics.

[26] Leutert M., Rodríguez-Mias R.A., Fukuda N.K., Villén J. (2019). R2-P2 rapid-robotic phosphoproteomics enables multidimensional cell signaling studies. Mol. Syst. Biol..

[27] Rosenberger G., Liu Y., Röst H.L., Ludwig C., Buil A., Bensimon A. (2017). Inference and quantification of peptidoforms in large sample cohorts by SWATH-MS. Nat. Biotechnol..

[28] Searle B.C., Lawrence R.T., MacCoss M.J., Villén J. (2019). Thesaurus: quantifying phosphopeptide positional isomers. Nat. Methods.

[29] Bekker-Jensen D.B., Bernhardt O.M., Hogrebe A., Martinez-Val A., Verbeke L., Gandhi T. (2020). Rapid and site-specific deep phosphoproteome profiling by data-independent acquisition without the need for spectral libraries. Nat. Commun..

[30] Marx H., Lemeer S., Schliep J.E., Matheron L., Mohammed S., Cox J. (2013). A large synthetic peptide and phosphopeptide reference library for mass spectrometry-based proteomics. Nat. Biotechnol..

[31] Barber K.W., Muir P., Szeligowski R.V., Rogulina S., Gerstein M., Sampson J.R. (2018). Encoding human serine phosphopeptides in bacteria for proteome-wide identification of phosphorylation-dependent interactions. Nat. Biotechnol..

[32] Gassaway B.M., Li J., Rad R., Mintseris J., Mohler K., Levy T. (2022). A multi-purpose, regenerable, proteome-scale, human phosphoserine resource for phosphoproteomics. Nat. Methods.

[33] Soste M., Hrabakova R., Wanka S., Melnik A., Boersema P., Maiolica A. (2014). A sentinel protein assay for simultaneously quantifying cellular processes. Nat. Methods.

[34] Parker B.L., Yang G., Humphrey S.J., Chaudhuri R., Ma X., Peterman S. (2015). Targeted phosphoproteomics of insulin signaling using data-independent acquisition mass spectrometry. Sci. Signal..

[35] Abelin J.G., Patel J., Lu X., Feeney C.M., Fagbami L., Creech A.L. (2016). Reduced-representation phosphosignatures measured by quantitative targeted MS capture cellular states and enable large-scale comparison of drug-induced phenotypes. Mol. Cell Proteomics.

[36] Stopfer L.E., Flower C.T., Gajadhar A.S., Patel B., Gallien S., Lopez-Ferrer D. (2021). High-density, targeted monitoring of tyrosine phosphorylation reveals activated signaling networks in human tumors. Cancer Res..

[37] Keshishian H., McDonald E.R., Mundt F., Melanson R., Krug K., Porter D.A. (2021). A highly multiplexed quantitative phosphosite assay for biology and preclinical studies. Mol. Syst. Biol..

[38] Hoopmann M.R., Kusebauch U., Palmblad M., Bandeira N., Shteynberg D.D., He L. (2020). Insights from the first phosphopeptide challenge of the MS resource pillar of the HUPO human proteome project. J. Proteome Res..

[39] Dele-Oni D.O., Christianson K.E., Egri S.B., Vaca Jacome A.S., DeRuff K.C., Mullahoo J. (2021). Proteomic profiling dataset of chemical perturbations in multiple biological backgrounds. Sci. Data.

[40] Whiteaker J.R., Sharma K., Hoffman M.A., Kuhn E., Zhao L., Cocco A.R. (2021). Targeted mass spectrometry-based assays enable multiplex quantification of receptor tyrosine kinase, MAP kinase, and AKT signaling. Cell Rep. Methods.

[41] Escher C., Reiter L., MacLean B., Ossola R., Herzog F., Chilton J. (2012). Using iRT, a normalized retention time for more targeted measurement of peptides. Proteomics.

[42] Duncan J.S., Whittle M.C., Nakamura K., Abell A.N., Midland A.A., Zawistowski J.S. (2012). Dynamic reprogramming of the kinome in response to targeted MEK inhibition in triple-negative breast cancer. Cell.

[43] Moritz A., Li Y., Guo A., Villén J., Wang Y., MacNeill J. (2010). Akt-RSK-S6 kinase signaling networks activated by oncogenic receptor tyrosine kinases. Sci. Signal..

[44] Manning B.D., Toker A. (2017). AKT/PKB signaling: navigating the network. Cell.

[45] Alessi D.R., Andjelkovic M., Caudwell B., Cron P., Morrice N., Cohen P. (1996). Mechanism of activation of protein kinase B by insulin and IGF-1. EMBO J..

[46] Frahm J.L., Howard B.E., Heber S., Muddiman D.C. (2006). Accessible proteomics space and its implications for peak capacity for zero-, one- and two-dimensional separations coupled with FT-ICR and TOF mass spectrometry. J. Mass Spectrom..

[47] Ivanov A.R., Colangelo C.M., Dufresne C.P., Friedman D.B., Lilley K.S., Mechtler K. (2013). Interlaboratory studies and initiatives developing standards for proteomics. Proteomics.

[48] Yue X., Schunter A., Hummon A.B. (2015). Comparing multistep immobilized metal affinity chromatography and multistep TiO2 methods for phosphopeptide enrichment. Anal. Chem..

[49] Amodei D., Egertson J., MacLean B.X., Johnson R., Merrihew G.E., Keller A. (2019). Improving precursor selectivity in data-independent acquisition using overlapping windows. J. Am. Soc. Mass Spectrom..

[50] Pino L.K., Searle B.C., Bollinger J.G., Nunn B., MacLean B., MacCoss M.J. (2020). The skyline ecosystem: informatics for quantitative mass spectrometry proteomics. Mass Spectrom. Rev..

[51] Eliuk S., Makarov A. (2015). Evolution of orbitrap mass spectrometry instrumentation. Annu. Rev. Anal. Chem..

[52] Pino L.K., Searle B.C., Huang E.L., Noble W.S., Hoofnagle A.N., MacCoss M.J. (2018). Calibration using a single-point external reference material harmonizes quantitative mass spectrometry proteomics data between platforms and laboratories. Anal. Chem..

[53] Huang T., Bruderer R., Muntel J., Xuan Y., Vitek O., Reiter L. (2020). Combining precursor and fragment information for improved detection of differential abundance in data independent acquisition. Mol. Cell Proteomics.

[54] Pino L.K., Baeza J., Lauman R., Schilling B., Garcia B.A. (2021). Improved SILAC quantification with data-independent acquisition to investigate bortezomib-induced protein degradation. J. Proteome Res..

[55] Mayer R.L., Matzinger M., Schmücker A., Stejskal K., Krššáková G., Berger F. (2022). Wide window acquisition and AI-based data analysis to reach deep proteome coverage for a wide sample range, including single cell proteomic inputs. bioRxiv.

[56] Villén J., Beausoleil S.A., Gerber S.A., Gygi S.P. (2007). Large-scale phosphorylation analysis of mouse liver. Proc. Natl. Acad. Sci. U. S. A..

[57] Schweiger R., Linial M. (2010). Cooperativity within proximal phosphorylation sites is revealed from large-scale proteomics data. Biol. Direct.

[58] Courcelles M., Bridon G., Lemieux S., Thibault P. (2012). Occurrence and detection of phosphopeptide isomers in large-scale phosphoproteomics experiments. J. Proteome Res..

[59] Beausoleil S.A., Villén J., Gerber S.A., Rush J., Gygi S.P. (2006). A probability-based approach for high-throughput protein phosphorylation analysis and site localization. Nat. Biotechnol..

[60] Olsen J.V., Blagoev B., Gnad F., Macek B., Kumar C., Mortensen P. (2006). Global, *in vivo*, and site-specific phosphorylation dynamics in signaling networks. Cell.

[61] Savitski M.M., Lemeer S., Boesche M., Lang M., Mathieson T., Bantscheff M. (2011). Confident phosphorylation site localization using the mascot delta score. Mol. Cell Proteomics.

[62] Taus T., Köcher T., Pichler P., Paschke C., Schmidt A., Henrich C. (2011). Universal and confident phosphorylation site localization using phosphoRS. J. Proteome Res..

[63] Fermin D., Walmsley S.J., Gingras A.-C., Choi H., Nesvizhskii A.I. (2013). LuciPHOr: algorithm for phosphorylation site localization with false localization rate estimation using modified target-decoy approach. Mol. Cell Proteomics.

[64] Suni V., Suomi T., Tsubosaka T., Imanishi S.Y., Elo L.L., Corthals G.L. (2018). SimPhospho: a software tool enabling confident phosphosite assignment. Bioinformatics.

[65] Joyce A., Searle B. (2023). Computational approaches to identify sites of phosphorylation. Authorea.

[66] Huang C.Y., Ferrell J.E. (1996). Ultrasensitivity in the mitogen-activated protein kinase cascade. Proc. Natl. Acad. Sci. U. S. A..

[67] Haystead T.A., Dent P., Wu J., Haystead C.M., Sturgill T.W. (1992). Ordered phosphorylation of p42mapk by MAP kinase kinase. FEBS Lett..

[68] Andreu-Pérez P., Esteve-Puig R., de Torre-Minguela C., López-Fauqued M., Bech-Serra J.J., Tenbaum S. (2011). Protein arginine methyltransferase 5 regulates ERK1/2 signal transduction amplitude and cell fate through CRAF. Sci. Signal..

[69] Palumbo A.M., Reid G.E. (2008). Evaluation of gas-phase rearrangement and competing fragmentation reactions on protein phosphorylation site assignment using collision induced dissociation-MS/MS and MS3. Anal. Chem..

[70] Nova I.C., Ritmejeris J., Brinkerhoff H., Koenig T.J.R., Gundlach J.H., Dekker C. (2022). Mapping phosphorylation post-translational modifications along single peptides with nanopores. bioRxiv.

