# Supplementary material for: A Multipathway Phosphopeptide Standard for Rapid Phosphoproteomics Assay Development
Source: Mol Cell Proteomics. 2023 Aug 30;22(10):100639. doi: 10.1016/j.mcpro.2023.100639 (PMC10561125; doi:10.1016/j.mcpro.2023.100639)
Supplement: Supplementary figures — Peptide observation consistency across labs. [file mmc1.docx]

Supplementary Figures for: A multipathway phosphopeptide standard for rapid phosphoproteomics assay development

**Authors:**

Brian C. Searle,^1,2,^* Allis Chien,^3^ Antonius Koller,^4^ David Hawke,^5^ Anthony W. Herren,^6^ Jenny Kim,^7^ Kimberly A. Lee,^8^ Ryan D. Leib,^3^ Alissa J. Nelson,^8^ Purvi Patel,^7^ Jian Min Ren,^8^ Paul M. Stemmer,^9^ Yiying Zhu,^8^ Benjamin A. Neely,^10^ Bhavin Patel^11^

**Affiliations:**

1) Department of Biomedical Informatics, The Ohio State University, Columbus, OH, USA
2) Pelotonia Institute for Immuno-Oncology, The Ohio State University Comprehensive Cancer Center, Columbus, OH, USA

3) Stanford University, Stanford CA, USA

4) YatiriBio, 6335 Nancy Ridge Drive, San Diego, CA, USA

5) BreakBio Corp, NY, NY, USA

6) UC Davis Genome Center, Proteomics Core, University of California Davis, Davis CA, USA

7) Columbia University Medical Center, NY, NY, USA

8) Cell Signaling Technology, Inc. Danvers, MA, USA

9) Wayne State University, Detroit, MI, USA

10) National Institute of Standards and Technology, Charleston, SC, USA

11) Thermo Fisher Scientific, Rockford, IL, USA

* Corresponding Author: [brian.searle@osumc.edu](mailto:brian.searle@osumc.edu), 590 BRT, 460 W 12th Ave,

Columbus, OH 43210

| 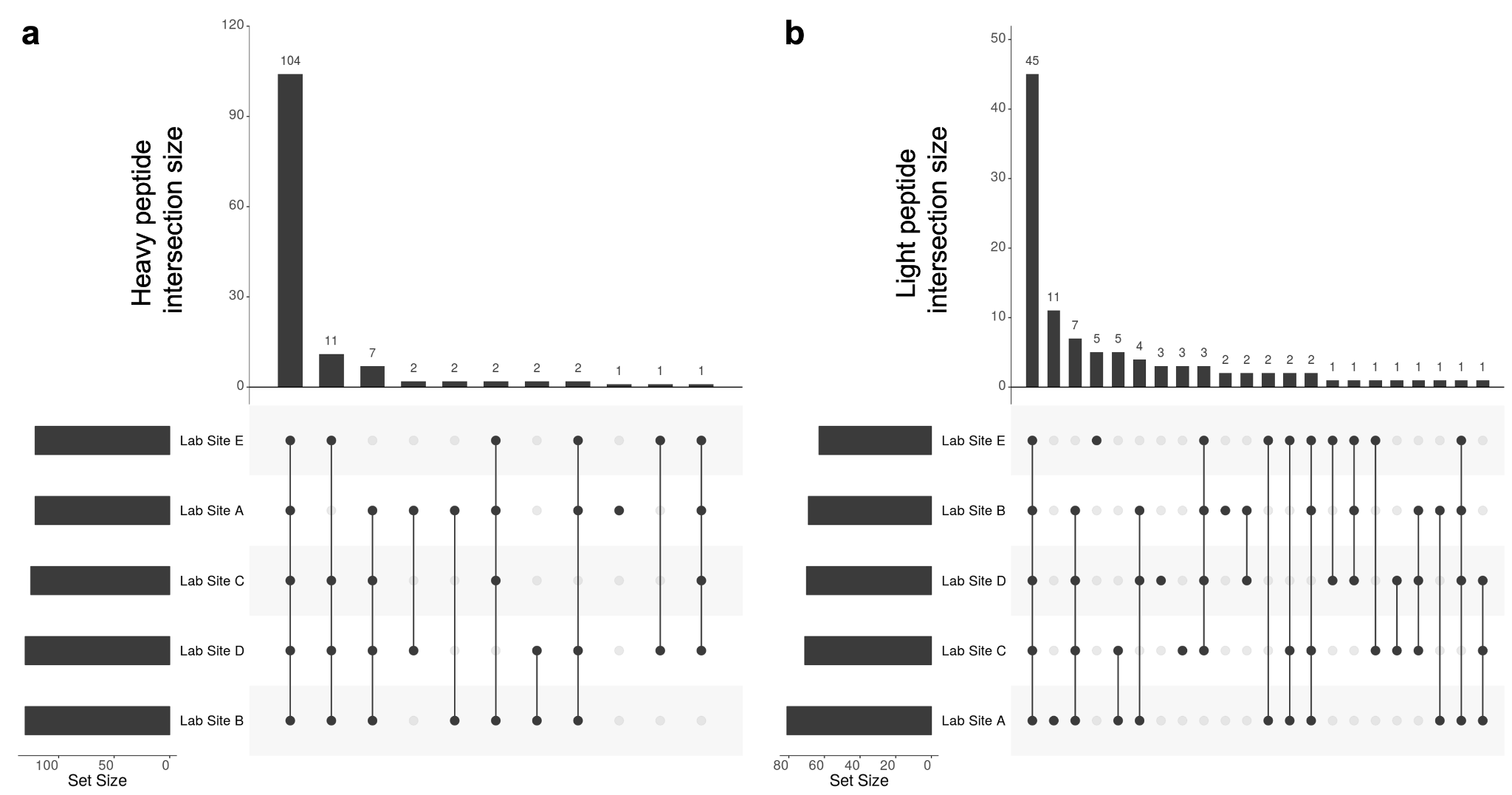 |
| --- |
| **Supplementary Figure 1: Peptide observation consistency across labs.** UpSet plots showing the intersection of heavy (**a**) and light (**b**) peptides observed by each lab site. |
